# Protective and therapeutic effects of nanoliposomal quercetin on acute liver injury in rats

**DOI:** 10.1186/s40360-020-0388-5

**Published:** 2020-02-14

**Authors:** Xiangyan Liu, Yang Zhang, Ling Liu, Yifeng Pan, Yu Hu, Pu Yang, Mingmei Liao

**Affiliations:** 1 Department of Breast Surgery, Xiangya Hospital, Central South University, Changsha, 410008 People’s Republic of China; 2 NHC Key Laboratory of Nanobiological Technology, Xiangya Hospital, Central South University, Changsha, 410008 People’s Republic of China; 3 Hepatobiliary and Enteric Surgery Center, Xiangya Hospital, Central South University, Changsha, 410008 People’s Republic of China; 4 Center for Experimental Medical Research, Third Xiangya Hospital, Central South University, Changsha, 410013 People’s Republic of China; 5 Department of Vascular Surgery, Xiangya Hospital, Central South University, Changsha, 410008 People’s Republic of China

**Keywords:** Quercetin, Liver injury, Liposome, Nanoparticles, Bioavailability

## Abstract

**Background:**

Quercetin, a pigment (flavonoid) found in many plants and foods, has good effects on protecting liver function but poor solubility and bioavailability in vivo. A drug delivery system can improve the accumulation and bioavailability of quercetin in liver. In this study, we used liposomal nanoparticles to entrap quercetin and evaluated its protective and therapeutic effects on drug-induced liver injury in rats.

**Methods:**

The nanoliposomal quercetin was prepared by a thin film evaporation-high pressure homogenization method and characterized by morphology, particle size and drug content. Acute liver injury was induced in rats by composite factors, including carbon tetrachloride injection, high-fat corn powder intake and ethanol drinking. After pure quercetin or nanoliposomal quercetin treatment, liver function was evaluated by detecting serum levels of glutamic-pyruvic transaminase (GPT), glutamic-oxal acetic transaminase (GOT) and direct bilirubin (DBIL). Histology of injured liver tissues was evaluated by hematoxylin and eosin staining.

**Results:**

On histology, liposomal nanoparticles loading quercetin were evenly distributed spherical particles. The nanoliposomal quercetin showed high bioactivity and bioavailability in rat liver and markedly attenuated the liver index and pathologic changes in injured liver tissue. With nanoliposomal quercetin treatment, the serum levels of GPT, GOT and DBIL were significantly better than treated with pure quercetin. Using liposomal nanoparticles to entrap quercetin might be an effective strategy to reduce hepatic injury and protect hepatocytes against damage.

**Conclusion:**

Liposomal nanoparticles may improve the solubility and bioavailability of quercetin in liver. Furthermore, nanoliposomal quercetin could effectively protect rats against acute liver injury and may be a new hepatoprotective and therapeutic agent for patients with liver diseases.

## Background

Hepatitis virus or toxin exposure leads to liver injury, eventually resulting in cirrhosis and liver cancer [1–3]. Therapy choices for liver injury are few. Quercetin, a pigment (flavonoid) found in many plants and foods such as wine, apple, onions, green tea, berries and some herbs, has wide bioactivity, including anti-oxidative, anti-fibrotic and anti-inflammatory effects [4–7]. Its protective effect on liver injury was previously identified [8–11]. However, quercetin has poor water solubility, so it cannot accumulate well in the liver.

Previous studies showed that various carriers such as chitosan or liposome could enhance the solubility of quercetin and improve its bioactivity for treating tumor [12] and diabetes [13, 14]. A drug delivery system is needed to improve the accumulation and bioavailability of quercetin in liver.

Here, we prepared liposomal nanoparticles loading quercetin and detected their effect on rat liver injury induced by complex factors, which is relatively close to the naturally occurring process. We identified the protective and therapeutic effects of nanoliposomal quercetin on drug-induced liver injury in rats. Liposomal nanoparticle-entrapped quercetin may be a potential therapeutic agent for hepatic diseases.

## Methods

### Materials

Refined soy lecithin Lipoid S100 was from Germany Lipoid GmbH (No.790495–1). Cholesterol was from Beijing Chemical Reagent Co. Quercetin was from Hunan nine modern Chinese Medicine Co. The hematoxylin and eosin (H&E) staining kit was from Shanghai Huyu Biological Technology Co. GPT/GOT ELISA kit was from Shanghai Ximei Biological Technology Co. Bilirubin assay kit was from Shanghai Hengyuan Biological Technology Co. Carbon tetrachloride was from Beijing Jinhuitaiya Chemical Reagent Co. All other reagents such as anhydrous alcohol, ether, and methanol were of analytical grade.

### Preparation and determination of nanoliposomal quercetin

Nanoliposomal quercetin was prepared by the thin film evaporation method as described [15]. Briefly, liposomes, containing with quercetin were prepared from phosphatidylcholine, phosphatidylserine and cholesterol (molar ratio: 5:1:1) by a modification of the thin film evaporation method. The ethanol in mixed solution was removed under reduced pressure at 37 °C by rotary evaporation to form a thin solid film. The lipid film was then hydrated with 5% glucose solution at 37 °C by rotation to form a light yellow suspension. With sonication and high-pressure homogenization, the suspension was then passed through a filter membrane (0.2 μm) to remove the nonincorporated drug, and yielded nanoliposomal quercetin. The morphology of the nanoparticles encapsulated with quercetin was evaluated by using a S-3400 N scanning electron microscope (SEM, Japan) and the size determination was detected by particle size analyzer (ZEN3600, Malvern, UK). The drug content was analyzed by high-performance liquid chromatography (HPLC). In the following experiments, the dose of nanoliposomal quercetin was based on the content of quercetin.

### Experimental animals

We obtained 40 healthy Sprague-Dawley (SD) rats with half male and half female, 80 to 120 g body weight, from the experimental animal department of Xiangya Medical College of Central South University, and rats were kept under lab conditions (20–25 °C, 50% relative humidity, 12-h light-dark cycle). The experiments were approved by the ethics committee of Xiangya Hospital of Central South University (reference: 201503239). All experimental animals were finally euthanized.Using pentobarbital sodium injection, intraperitoneal injection of the drug 150–200 mg / kg can make the animal stop breathing. Since the experimental rats weighed 80–120 g, 200 mg/kg sodium pentobarbital was used to achieve euthanasia in all mice.

### Treatments in hepatic-injured rat models

Rats were randomly divided into 4 groups (10 rats per group): normal control and 3 groups of hepatic-injured rats that received saline, quercetin, or nanoliposomal quercetin. Liver injury was induced by composite factors [16–19], including carbon tetrachloride injection, high-fat corn powder intake and ethanol drinking. First, rats were injected subcutaneously with a castor oil solution containing 40% carbon tetrachloride at 0.5 mL per 100 g body weight, then 0.3 mL per 100 g body weight every 3 days for 13 times. Simultaneously, rats were fed high-fat corn powder (79.5% pure corn powder with 10% lard and 0.5% cholesterol) and drank 10% ethanol as the only liquid every day. After the first subcutaneous injection, all groups were injected intraperitoneally with 0.9% normal saline for control and saline-treated rats and pure quercetin and nanoliposomal quercetin at 5 mg/100 g body weight in 0.2 mL once a day for 1 week, then every 2 days for 4 weeks for quercetin- and nanoliposomal quercetin-treated rats. About 6 weeks later, all rats were killed after anesthesia, and blood and liver samples were collected for further analysis. At 1 day after the last drug injection, 2 mL blood per rat was obtained from the inferior vena cava for liver function detection, including serum levels of glutamic-pyruvic transaminase (GPT), glutamic-oxal acetic transaminase (GOT) and direct bilirubin (DBIL). The livers of all groups were promptly harvested, weighed and fixed in 10% formalin, then processed for histology. The liver index was calculated by the ratio of liver weight and body weight (Liver index = liver weight/body weight× 100).

### Histology

Liver tissues were fixed in 10% formalin overnight at room temperature, then underwent gradient alcohol dehydration, embedding in paraffin, and sectioning at a 5 μm thick. Sections were stained by H&E staining and observed under a microscope for gross pathology evaluation.

### Statistical analysis

Data are reported as mean ± SEM. SPSS for Windows 18.0, GraphPad Prism 7 and Microsoft Office 2010 Excel were used for data analysis. One-way ANOVA were performed, when appropriate, with Holm–Sidak analysis being used for post hoc tests. *P* < 0.05 was considered statistically significant.

## Results

### Characteristies of nanoliposomal quercetin

**Fig. 1:**
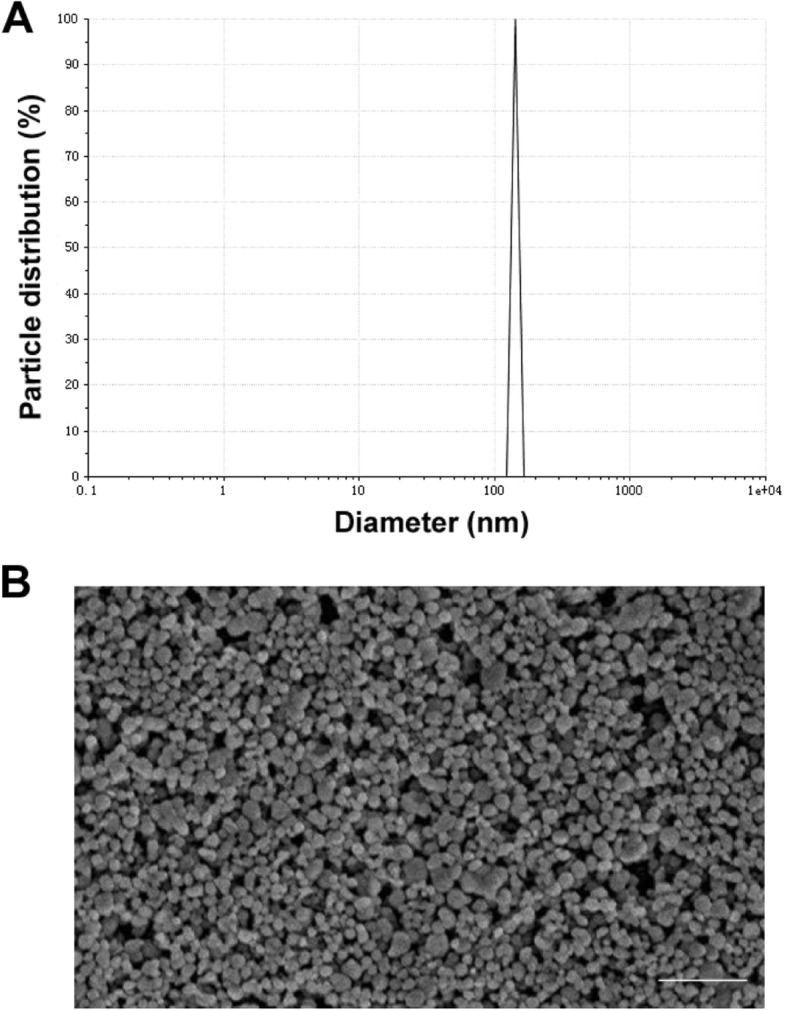
Characteristics of nanoliposomal quercetin. **a** Size distribution of nanoliposomal quercetin; **b** Scanning electron microscopy image of nanoliposomal quercetin. Scale bar: 1 μm

Quercetin, present in fruits and vegetables, is one of the most common flavonoidal compounds, with non-lethal, non-carcinogenic, non-teratogenic and non-mutation effects in humans. However, quercetin is not soluble in water and has poor bioavailability in liver. Nanoparticles can change the transport mechanism of the cell membrane and increase the accumulation of quercetin in the liver to improve its bioavailability [9, 13]. As shown in Fig. 1b, the nanoparticles were evenly distributed spherical particles. The size of nanoliposomal quercetin was 142 ± 19 nm (Fig. 1a). The drug loading was 5.08 ± 0.17 mg/mL. The nanoparticles were stable and had no stratification after being placed at room temperature for 3 months, and were kept in the form of freeze-dried powder for a long time. Before experimental use, the prepared powder was well resuspended in 0.9% normal saline using ultrasound treatment to obtain a suspension of nanoliposomal quercetin. The listed dose of liposomal quercetin is based on the content of quercetin.

### Effect of quercetin treatments on body weight and liver index of the hepatic-injured rats

**Fig. 2:**
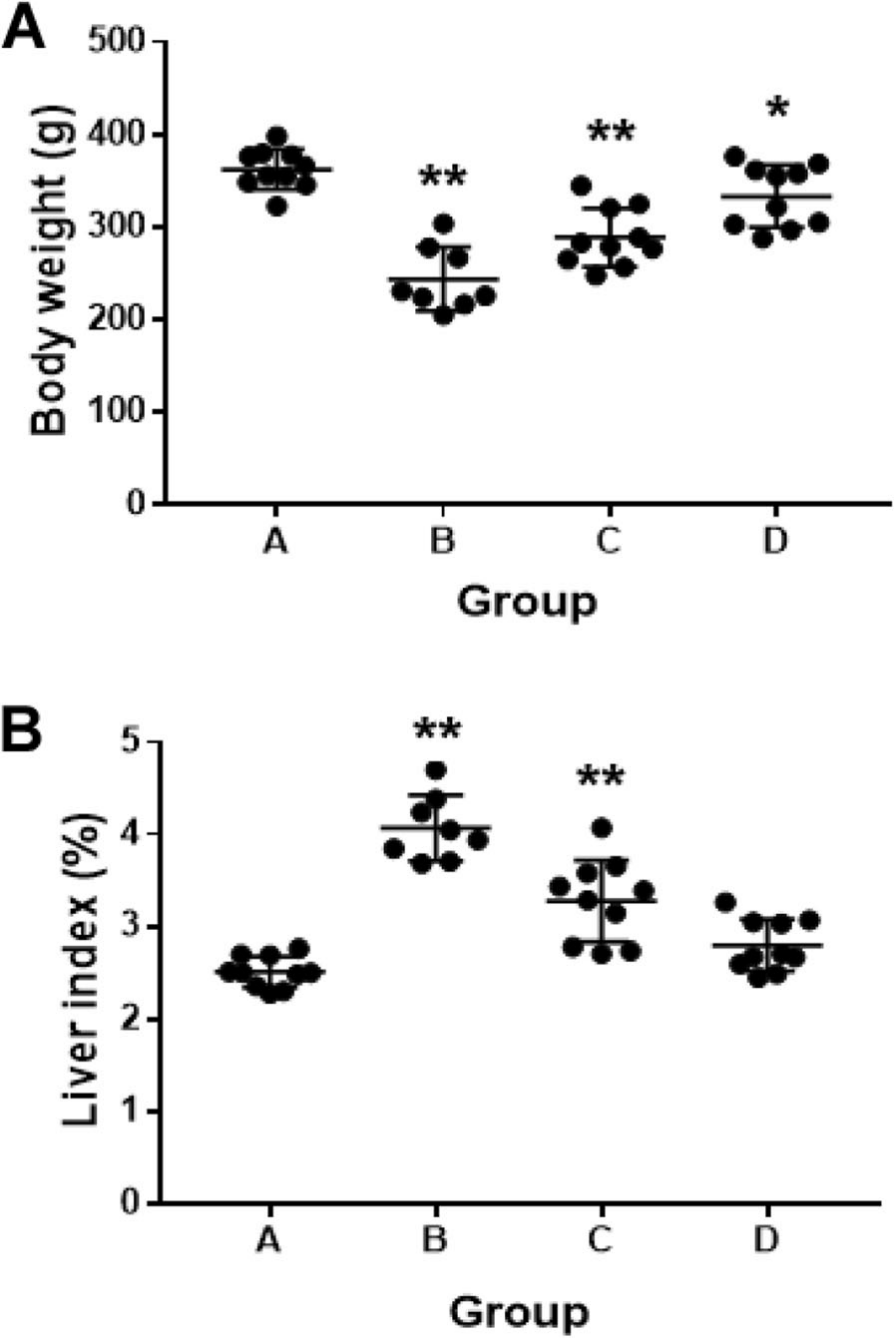
Body weight and liver index of the rats after treatments. **a** (A) Normal control; (B-D) hepatic-injured rats with **b** B) saline treatment; C) quercetin treatment; and D) nanoliposomal quercetin treatment. Data are mean ± SEM (*n* = 8–10). *P < 0.05, **P < 0.01, compared with the normal control group, by *t* test

As compared with the other groups, hepatic-injured rats with saline treatment showed significantly decreased food intake, body weight and activity accompanied by poor light reactions. Furthermore, two hepatic-injured rats in the saline treatment group died of liver failure before tissue harvesting. As shown in Fig. 2a, simultaneous treatment of pure quercetin and nanoliposomal quercetin significantly increased the body weight of hepatic-injured rats when compared with the hepatic-injured rats with saline treatment (*P* < 0.01). However, the liver weight of hepatic-injured rats with saline treatment was heavier with fat accumulation in liver than the other groups (9.85 ± 0.32 g in saline treatment group, 9.12 ± 0.26 g in normal control group, 9.36 ± 0.15 g in pure quercetin group and 9.27 ± 0.14 g in nanoliposomal quercetin group, respectively). As shown in Fig. 2b, the liver index of hepatic-injured rats with saline treatment was 4.07 ± 0.13%, and quercetin treatment can markedly decreased the liver index of hepatic-injured rats (3.28 ± 0.14% in pure quercetin group and 2.80 ± 0.09% in nanoliposomal quercetin group). Compared with the pure quercetin group, nanoliposomal quercetin was more effective on decreasing liver index of hepatic-injured rats (*P* = 0.005). Moreover, the liver index showed no significant difference in nanoliposomal quercetin group with the normal control group (*P* > 0.05). The results indicate that the protective effect of nanoliposomal quercetin in the injured liver was stronger than that of pure quercetin.

### Effect of nanoliposomal quercetin on serum liver function of experimental rats

To determine the effect of nanoliposomal quercetin on liver function, we detected serum levels of GPT, GOT and DBIL by using GPT/GOT ELISA kit and bilirubin assay kit. As shown in Table 1, GPT, GOT and DBIL values for hepatic-injured rats with saline treatment were higher than for control rats (*P* < 0.001). With simultaneous treatment of pure quercetin and nanoliposomal quercetin in rats with hepatic injury, the values of GPT, GOT and DBIL markedly decreased as compared with saline treatment. Liver function did not differ between nanoliposomal quercetin and control treatment (*P* > 0.05), which indicates the hepatoprotective and treatment effects of nanoliposomal quercetin on liver function of rats.

**Table 1:** Effect of nanoliposomal quercetin on liver function in rats

Group	GPT(μ/L)	GOT(μ/L)	DBIL(μmol/L)
Normal control	33.63 ± 2.87**	59.88 ± 7.07**	0.21 ± 0.03**
Saline treatment	494.88 ± 6.53	765.50 ± 9.79	2.18 ± 0.06
Quercetin treatment	67.63 ± 6.49**	124.88 ± 14.76**	0.56 ± 0.07**
Nanoliposomal quercetin treatment	42.88 ± 3.39**^△△#^	78.75 ± 8.64**^△#^	0.28 ± 0.04**^△△#^

Note. Data are mean ± SEM (*n* = 8–10). **P < 0.01, compared with Group B; ^△^*P* < 0.05, ^△△^*P* < 0.01, compared with Group C; ^#^*P* > 0.05, compared with Group A, by *t* test

*GPT* glutamic-pyruvic transaminase, *GOT* glutamic-oxal acetic transaminase, *DBIL* direct bilirubin

### Effect of nanoliposomal quercetin on histopathology of injuried liver

**Fig. 3:**
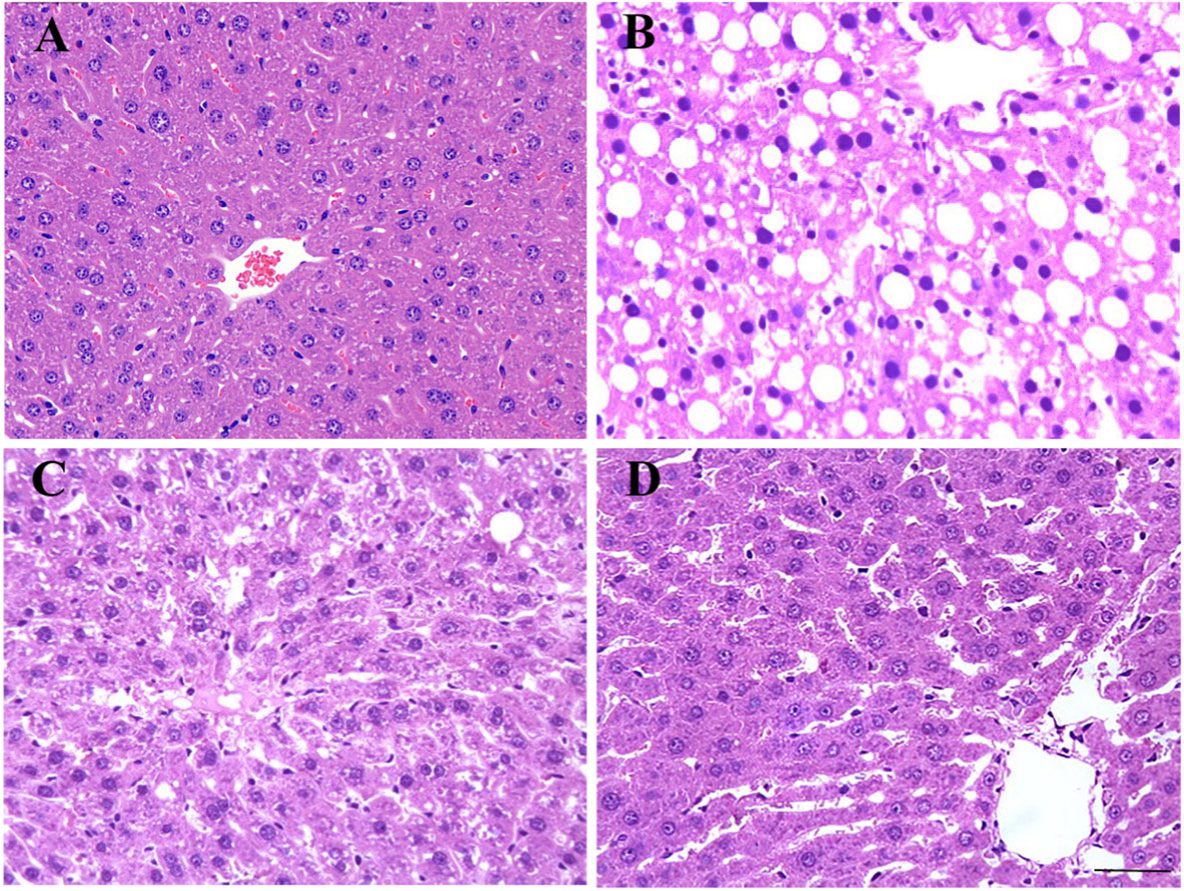
Effect of nanoliposomal quercetin on histopathological changes in rat liver tissues. (Scale bar: 50 μm; HE staining). **a** Normal control; **b**-**d** hepatic-injured rats with **b** saline treatment; **c** quercetin treatment; and **d** nanoliposomal quercetin treatment

Although the liver index and liver function are important indexes to reflect the severity of liver injury, the gold standard is still histopathological examination. It is always used to diagnose liver inflammation activity, evaluate the degree of liver injury and determine drug efficacy. To evaluate the effect of nanoliposomal quercetin on histopathology of the injured liver, liver sections from all four groups were stained with H&E. In control rats, the structures of hepatic lobules were clear and intact (Fig. 3a). Hepatocytes were arranged around the central vein in a single line and had a radial shape with no lesions. Hepatic sinusoids, interlobular arteries, interlobular veins and interlobular bile ducts in portal areas were normal. However, in hepatic-injured rats with saline treatment, hepatic lobules lost their normal structure and boundaries were not clear. Most hepatic cords and hepatic sinusoids resolved and disappeared (Fig. 3b). Liver sinuses around the hepatic lobules were narrowed significantly, accompanied by focal fibrosis and collagen fiber hyperplasia in portal areas. In addition, many inflammatory cells were visible. With the formation of lipid droplets and diffuse vacuolar degeneration, fatty degeneration and necrotic hepatocytes were widespread. Parts of hepatocytes showed typical ballooning degeneration, nuclear condensation, and shrinkage of the nuclear membrane and widespread death. Thus, the rat model of complex hepatic injury was successfully established.

In hepatic-injured rats with quercetin and nanoliposomal quercetin treatment, the pathologic changes of liver tissues with saline treatment were attenuated (Fig. 3c-d). As compared with saline treatment in hepatic-injured rats, nanoliposomal quercetin treatment showed intact structures of the hepatic lobule and liver cells, accompanied by resolved fatty change, no obvious fibrosis formation and no obvious infiltrates of interstitial inflammatory cells (Fig. 3d), which still could be found in liver tissues of pure quercetin-treated rats (Fig. 3c). Our data indicate that treatment with nanoliposomal quercetin could reduce hepatic injury and protect hepatocytes against damage effectively.

## Discussion

Many kinds of factors including biological damage such as with hepatitis virus [17], chemical damage such as with drugs [18, 19], and physical damage such as with liver transplantationcan [20, 21] lead to liver injury. The use of carbon tetrachloride [22] and other composite factors such as a high-fat, high-cholesterol, and low-protein diet as well as alcohol drinking can induce liver injury models in animals, with pathological results very similar to human chronic liver diseases. Quercetin is a hepatoprotective drug [23] but with poor solubility and bioavailability in liver [14, 24]. In this study, we used liposomal nanoparticles as the drug carrier loading quercetin to improve the solubility and bioavailability of quercetin in liver and protect rats against acute liver injury.

Quercetin, an organic flavone, presents no toxic effect at a oral dose up to 2000 mg/kg body weight in rats [25, 26]. Moreover, it possesses anticancer effects [27, 28], prevents the development of hepatic fibrosis [29, 30], and reduces toxicant-induced liver injury [8, 11]. However, the limited solubility of quercetin in water and poor bioavailability in liver present a major problem for its administration as a chemopreventer. Our study used a drug carrier, liposome, to entrap quercetin. The main component of liposomes is phospholipids, which has a typical feature of both hydrophilicity and hydrophobicity. It can be used as a drug carrier to bind with a variety of drugs and has many advantages, such as good biocompatibility, no toxic effects and good bioavailability [9, 13]. Furthermore, we nanoformulated the liposome-entrapped quercetin compound to improve the utilization of quercetin and targeting to the liver [13] quercetin in the liver to improve its bioavailability [31]. As shown in Fig. 1, the nanoparticles were evenly distributed spherical particles with the size about 142 nm. Quercetin was located in the middle of lipid bilayer with the drug loading about 5.08 mg/mL. The nanoparticles are stable in the form of freeze-dried powder for a long time storage and resuspend in 0.9% normal saline before experimental use. All the components of nanoparticles can be easily biodegradable and amenable to physiological excretion.

Under physiological conditions, the content of transaminase in liver cells is about 100 times that in peripheral blood. The hepatic cellular membranes can be damaged by various pathogenic factors, with some enzymes secreting to peripheral blood. Thus, transaminases (GPT and GOT) in serum are sensitive indicators of hepatocyte damage and can reflect the severity of liver injury to a certain extent [32]. The liver is the main organ of bilirubin metabolism, and liver cells are the only cells that can form direct bilirubin (DBIL). Therefore, the level of serum bilirubin, especially DBIL, can comprehensively reflect the functional status of liver cells including their intake, transport, binding and excretion. We use carbon tetrachloride and other composite factors such as a high-fat, high-cholesterol, and low-protein diet as well as alcohol drinking, which can induce liver injury in animalswith pathological results very similar to human chronic liver diseases.

As compared with the control group, hepatic-injured rats showed significantly decreased food intake, body weight and activity accompanied by poor light reactions. With the treatment of pure quercetin and nanoliposomal quercetinin in rats with hepatic injury, the body weight of hepatic-injured rats was increased significantly while the liver index and the values of GPT, GOT and DBIL markedly decreased as compared with saline treatment (Fig. 2 and Table 1). Moreover, Histopathological analysis (Fig. 3) in hepatic-injured rats indicates that nanoliposomal quercetin could reduce hepatic injury and protect hepatocytes against damage effectively. Interestingly, nanoliposomal quercetin was more effective on decreasing liver index and interstitial inflammatory infiltration of hepatic-injured rats than pure quercetin, which indicated that the protective effect of nanoliposomal quercetin in the injured liver was stronger than that of pure quercetin.

Further studies should define the therapeutic efficacy of the nanoliposome-entrapped quercetin compound and detect onset time, organ distribution and the pharmacokinetics of quercetin in vivo in order to provide a more efficacy profile for this promising therapeutic agent.

## Conclusions

Liposomal nanoparticles may improve the solubility and bioavailability of quercetin in liver. Furthermore, nanoliposomal quercetin could effectively protect rats against acute liver injury and may be a new hepatoprotective and therapeutic agent for patients with liver diseases.

## Data Availability

The datasets used and/or analyzed during the current study are available from the corresponding author on reasonable request.

## Abbreviations

ANOVA: Analysis of Variance
DBIL: Direct bilirubin
GOT: Glutamic-oxal acetic transaminase
GPT: Glutamic-pyruvic transaminase
H&E: Hematoxylin and eosin
SD: Sprague-Dawley

## References

[1] Tsochatzis EA, Bosch J, Burroughs AK (2014). Liver cirrhosis. Lancet.

[2] Arzumanyan A, Reis HM, Feitelson MA (2013). Pathogenic mechanisms in HBV- and HCV-associated hepatocellular carcinoma. Nat Rev Cancer.

[3] Moradpour D, Blum HE (2005). Pathogenesis of hepatocellular carcinoma. Eur J Gastroenterol Hepatol.

[4] Shokoohinia Y, Rashidi M, Hosseinzadeh L, Jelodarian Z (2015). Quercetin-3-O-beta-D-glucopyranoside, a dietary flavonoid, protects PC12 cells from H(2)O(2)-induced cytotoxicity through inhibition of reactive oxygen species. Food Chem.

[5] Sonoki H, Sato T, Endo S, Matsunaga T, Yamaguchi M, Yamazaki Y, Sugatani J, Ikari A (2015). Quercetin decreases Claudin-2 expression mediated by up-regulation of microRNA miR-16 in lung adenocarcinoma A549 cells. Nutrients.

[6] Pinelo M, Manzocco L, Nunez MJ, Nicoli MC (2004). Interaction among phenols in food fortification: negative synergism on antioxidant capacity. J Agric Food Chem.

[7] Hatahet T, Morille M, Shamseddin A, Aubert-Pouessel A, Devoisselle JM, Begu S (2017). Dermal quercetin lipid nanocapsules: influence of the formulation on antioxidant activity and cellular protection against hydrogen peroxide. Int J Pharm.

[8] Kemelo MK, Pierzynova A, Kutinova Canova N, Kucera T, Farghali H (2017). The involvement of sirtuin 1 and heme oxygenase 1 in the hepatoprotective effects of quercetin against carbon tetrachloride-induced sub-chronic liver toxicity in rats. Chem Biol Interact.

[9] Ghosh A, Mandal AK, Sarkar S, Das N (2011). Hepatoprotective and neuroprotective activity of liposomal quercetin in combating chronic arsenic induced oxidative damage in liver and brain of rats. Drug Deliv.

[10] Ji LL, Sheng YC, Zheng ZY, Shi L, Wang ZT (2015). The involvement of p62-Keap1-Nrf2 antioxidative signaling pathway and JNK in the protection of natural flavonoid quercetin against hepatotoxicity. Free Radic Biol Med.

[11] Afifi NA, Ibrahim MA, Galal MK (2018). Hepatoprotective influence of quercetin and ellagic acid on thioacetamide-induced hepatotoxicity in rats. Can J Physiol Pharmacol.

[12] Zhuo H, Zheng B, Liu J, Huang Y, Wang H, Zheng D, Mao N, Meng J, Zhou S, Zhong L, Zhao Y (2018). Efficient targeted tumor imaging and secreted endostatin gene delivery by anti-CD105 immunoliposomes. J Exp Clin Cancer Res.

[13] Wang G, Wang JJ, Yang GY, Du SM, Zeng N, Li DS, Li RM, Chen JY, Feng JB, Yuan SH, Ye F (2012). Effects of quercetin nanoliposomes on C6 glioma cells through induction of type III programmed cell death. Int J Nanomedicine.

[14] Mukhopadhyay P, Maity S, Mandal S, Chakraborti AS, Prajapati AK, Kundu PP (2018). Preparation, characterization and in vivo evaluation of pH sensitive, safe quercetin-succinylated chitosan-alginate core-shell-corona nanoparticle for diabetes treatment. Carbohydr Polym.

[15] Zhang YD, Wang JW, Liu XY, Zhao ZY, Zhang LH, Long B (2007). Study on distribution of liposome nanoparticles loaded quercetin in rats. Chin Med Eng.

[16] Zhang HY, Han DW, Zhao ZF, Liu MS, Wu YJ, Chen XM, Ji C (2007). Multiple pathogenic factor-induced complications of cirrhosis in rats: a new model of hepatopulmonary syndrome with intestinal endotoxemia. World J Gastroenterol.

[17] Billerbeck E, Wolfisberg R, Fahnoe U, Xiao JW, Quirk C, Luna JM, Cullen JM, Hartlage AS, Chiriboga L, Ghoshal K, Lipkin WI, Bukh J, Scheel TKH, Kapoor A, Rice CM (2017). Mouse models of acute and chronic hepacivirus infection. Science.

[18] Cubero FJ, Zoubek ME, Hu W, Peng J, Zhao G, Nevzorova YA, Al Masaoudi M, Bechmann LP, Boekschoten MV, Muller M, Preisinger C, Gassler N, Canbay AE, Luedde T, Davis RJ, Liedtke C, Trautwein C (2016). Combined activities of JNK1 and JNK2 in hepatocytes protect against toxic liver injury. Gastroenterology.

[19] Wang L, Zhang W, Ge CH, Yin RH, Xiao Y, Zhan YQ, Yu M, Li CY, Ge ZQ, Yang XM (2017). Toll-like receptor 5 signaling restrains T-cell/natural killer T-cell activation and protects against concanavalin A-induced hepatic injury. Hepatology.

[20] M.W. Wang X, Fang C, Tian S, Zhu X, Yang L, Huang Z, Li H, Dusp14 protects against hepatic ischemia-reperfusion injury via Tak1 suppression. J Hepatol. 2017;S0168-8278(17):32275-4. 10.1016/j.jhep.2017.08.032 28887166

[21] Nakao T, Ono Y, Dai H, Nakano R, Perez-Gutierrez A, Camirand G, Huang H, Geller DA, Thomson AW (2019). DNAX activating protein of 12 kDa/triggering receptor expressed on myeloid cells 2 expression by mouse and human liver dendritic cells: functional implications and regulation of liver ischemia-reperfusion injury. Hepatology.

[22] Qiao H, Zhou Y, Qin X, Cheng J, He Y, Jiang Y (2018). NADPH oxidase signaling pathway mediates Mesenchymal stem cell-induced inhibition of hepatic stellate cell activation. Stem Cells Int.

[23] Jia FF, Tan ZR, McLeod HL, Chen Y, Ou-Yang DS, Zhou HH (2016). Effects of quercetin on pharmacokinetics of cefprozil in Chinese-Han male volunteers. Xenobiotica.

[24] Tzankova V, Aluani D, Kondeva-Burdina M, Yordanov Y, Odzhakov F, Apostolov A, Yoncheva K (2017). Hepatoprotective and antioxidant activity of quercetin loaded chitosan/alginate particles in vitro and in vivo in a model of paracetamol-induced toxicity. Biomed Pharmacother.

[25] Gupta V, Sharma R, Bansal P, Kaur G (2018). Bioactivity-guided isolation of potent anxiolytic compounds from leaves of Citrus paradisi. Ayu.

[26] Dra LA, Sellami S, Rais H, Aziz F, Aghraz A, Bekkouche K, Markouk M, Larhsini M (2019). Antidiabetic potential of Caralluma europaea against alloxan-induced diabetes in mice. Saudi J Biol Sci.

[27] Chen FY, Cao LF, Wan HX, Zhang MY, Cai JY, Shen LJ, Zhong JH, Zhong H (2015). Quercetin enhances adriamycin cytotoxicity through induction of apoptosis and regulation of mitogen-activated protein kinase/extracellular signal-regulated kinase/c-Jun N-terminal kinase signaling in multidrug-resistant leukemia K562 cells. Mol Med Rep.

[28] Daglioglu C (2017). Enhancing tumor cell response to multidrug resistance with pH-sensitive Quercetin and doxorubicin conjugated multifunctional nanoparticles. Colloids Surf B Biointerfaces.

[29] Li X, Jin Q, Yao Q, Xu B, Li L, Zhang S, Tu C (2018). The flavonoid Quercetin ameliorates liver inflammation and fibrosis by regulating hepatic macrophages activation and polarization in mice. Front Pharmacol.

[30] Wu L, Zhang Q, Mo W, Feng J, Li S, Li J, Liu T, Xu S, Wang W, Lu X, Yu Q, Chen K, Xia Y, Lu J, Xu L, Zhou Y, Fan X, Guo C (2017). Quercetin prevents hepatic fibrosis by inhibiting hepatic stellate cell activation and reducing autophagy via the TGF-beta1/Smads and PI3K/Akt pathways. Sci Rep.

[31] Varshosaz J, Jafarian A, Salehi G, Zolfaghari B (2014). Comparing different sterol containing solid lipid nanoparticles for targeted delivery of quercetin in hepatocellular carcinoma. J Liposome Res.

[32] Karimi-Khouzani O, Heidarian E, Amini SA (2017). Anti-inflammatory and ameliorative effects of gallic acid on fluoxetine-induced oxidative stress and liver damage in rats. Pharmacol Rep.

